# Supplementary material for: Hmgb2 improves astrocyte to neuron conversion by increasing the chromatin accessibility of genes associated with neuronal maturation in a proneuronal factor-dependent manner
Source: Genome Biol. 2025 Apr 17;26:100. doi: 10.1186/s13059-025-03556-z (PMC12007351; doi:10.1186/s13059-025-03556-z)
Supplement: Supplementary file 1 — Additional File 1: Supplementary Figures. This file contains all supplementary figures [file 13059_2025_3556_MOESM1_ESM.pdf]

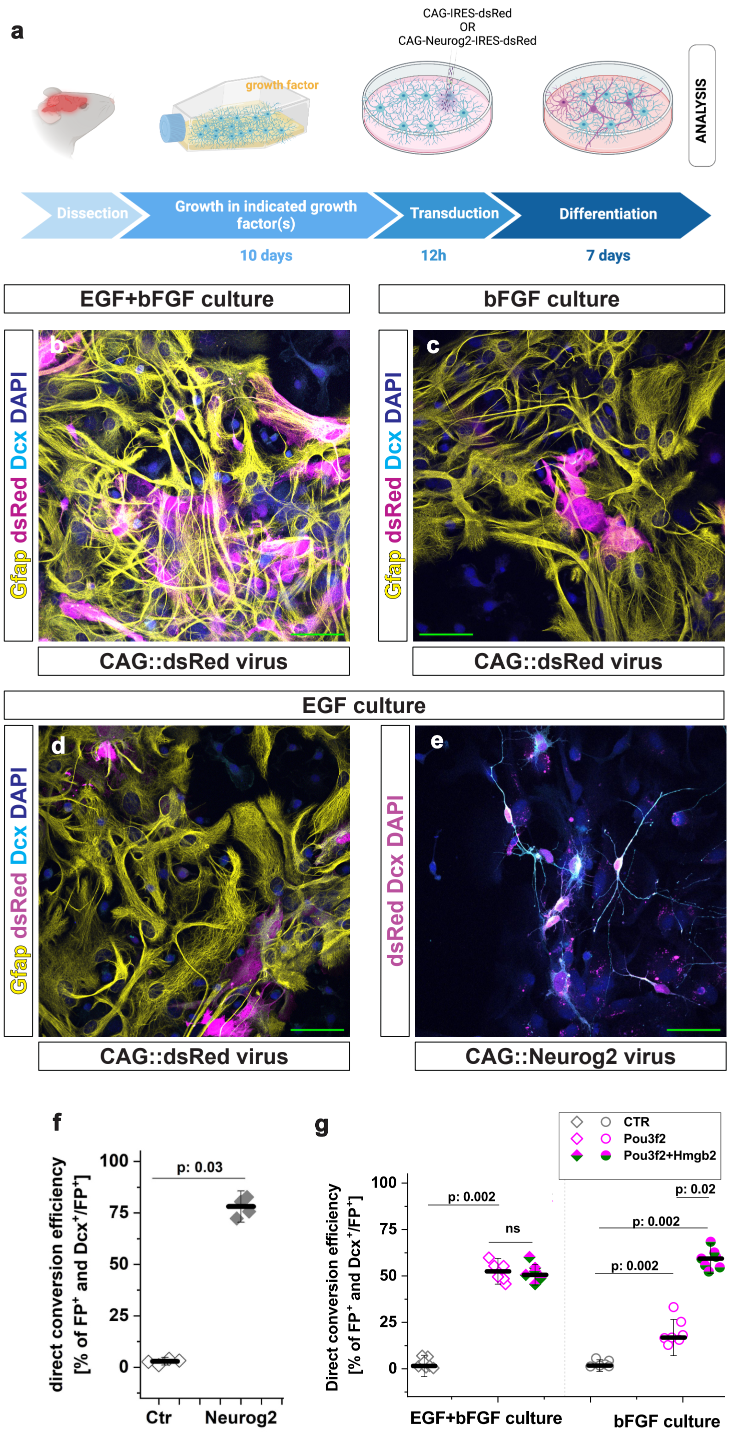

Figure S1. Growth conditions define the direct conversion rate.
(**a**) Scheme depicting the experimental paradigm used for astrocyte to neuron conversion. (**b-e**) Micrographs depicting the fate of transduced cells after control viral transduction in EGF+bFGF (b), bFGF (c), EGF (d) culture and Neurog2 overexpression in EGF culture (e) 7 days after viral transduction. Scale bars: 50 µm. (**f**, **g**) Dot plots showing direct conversion efficacy of Neurog2 overexpression in EGF culture (f) as well as Pou2f2, and Pou3f2+Hmgb2 overexpression in EGF+bFGF and bFGF culture (g). Data are shown as median±IQR; single dots represent independent biological replicates. Significance was tested with two-tailed Mann-Whitney test. Abbreviations: FP, fluorescent protein.

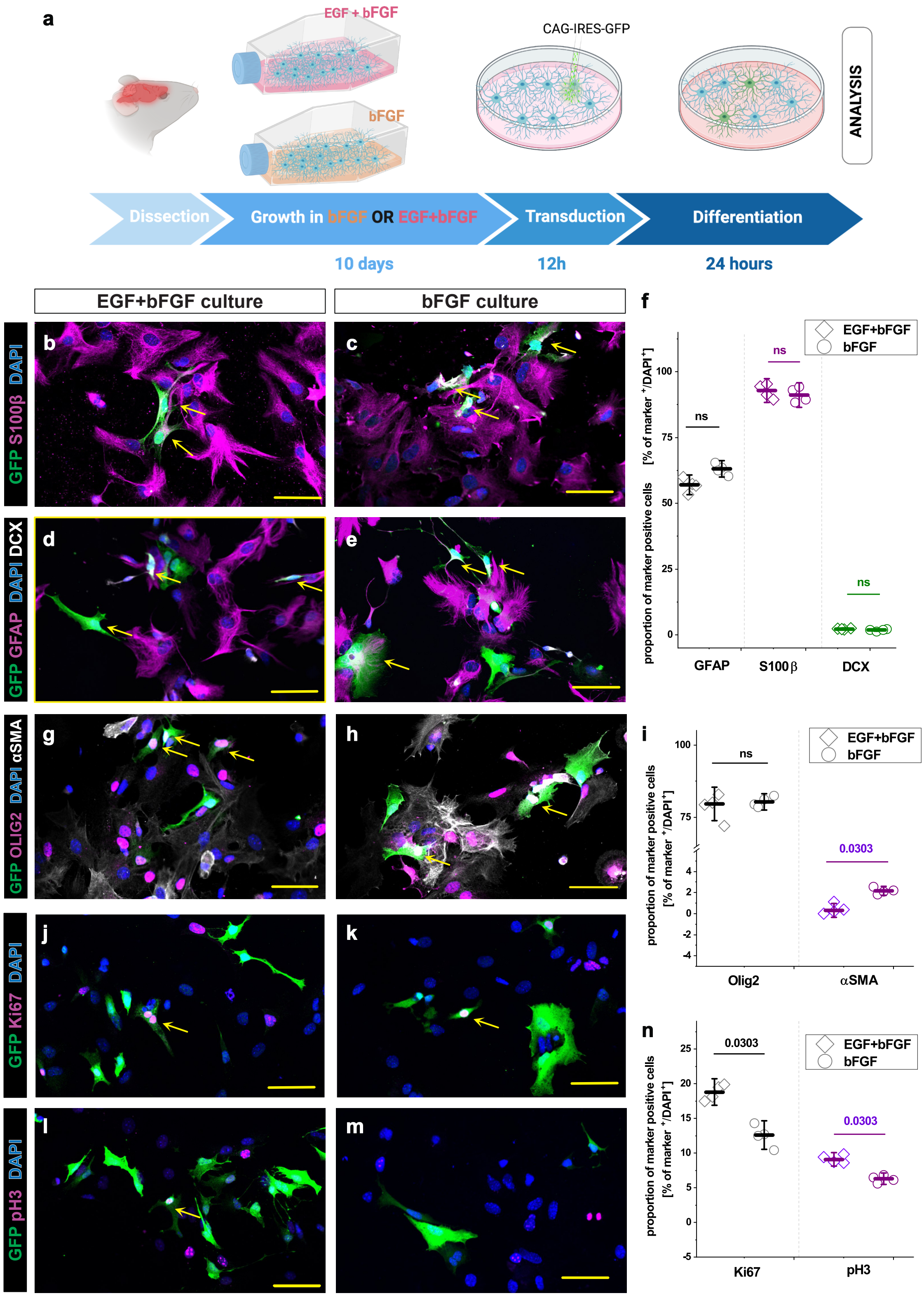

Figure S2: Characterization of the starting population in EGF+bFGF and bFGF culture.
(**a**) Scheme depicting the experimental paradigm used to characterize initially transduced cells. (**b**, **c**, **d**, **e**, **g**, **h**, **j**, **k**, **l**, **m**) Micrographs illustrating identity assessment of control virally transduced cells 24 h after transduction. Yellow arrows indicate identity marker positive transduced, GFP-positive cells. Scale bars: 50 µm. (**f, i, n**) Dot plots showing the proportion of transduced cells with the indicated identity. Data are shown as median±IQR; single dots represent independent biological replicates. Significance was tested with two-tailed Mann-Whitney test.

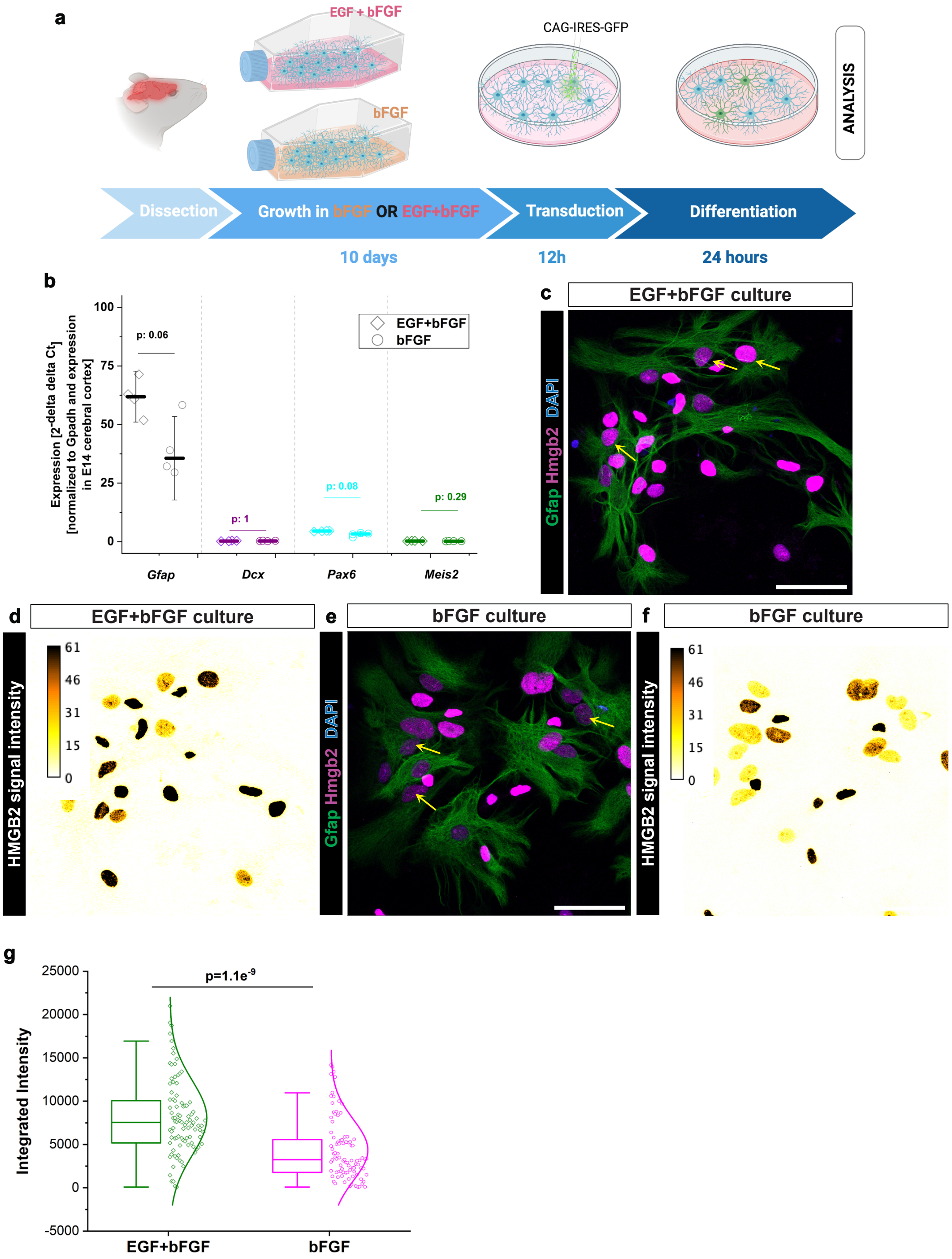

Figure S3: Expression of progenitor genes and Hmgb2 in astrocytes cultured with different growth factors.

(**a**) Scheme depicting the experimental paradigm used to address the expression level of progenitor genes and Hmgb2. (**b**) Dot plot depiction expression levels of progenitor genes and Gfap in astrocyte culture normalized to the expression in E14 cerebral cortex. Data are shown as median±IQR; single dots represent independent biological replicates. Significance was tested with two-tailed Mann-Whitney test. (**b-f**) Micrographs illustrating the expression of Hmgb2 in astrocytes grown with EGF+bFGF (c, d) and bFGF (e, f). d, f are pseudo colored images depicting expression level of Hmgb2 in single cells. Scale bar in b-f: 50 µm. (**g**) Dot plot showing the expression level of Hmgb2 in single cells. Single dots represent single cells from 4 independent biological replicates. Significance was tested with two-tailed Mann-Whitney test.

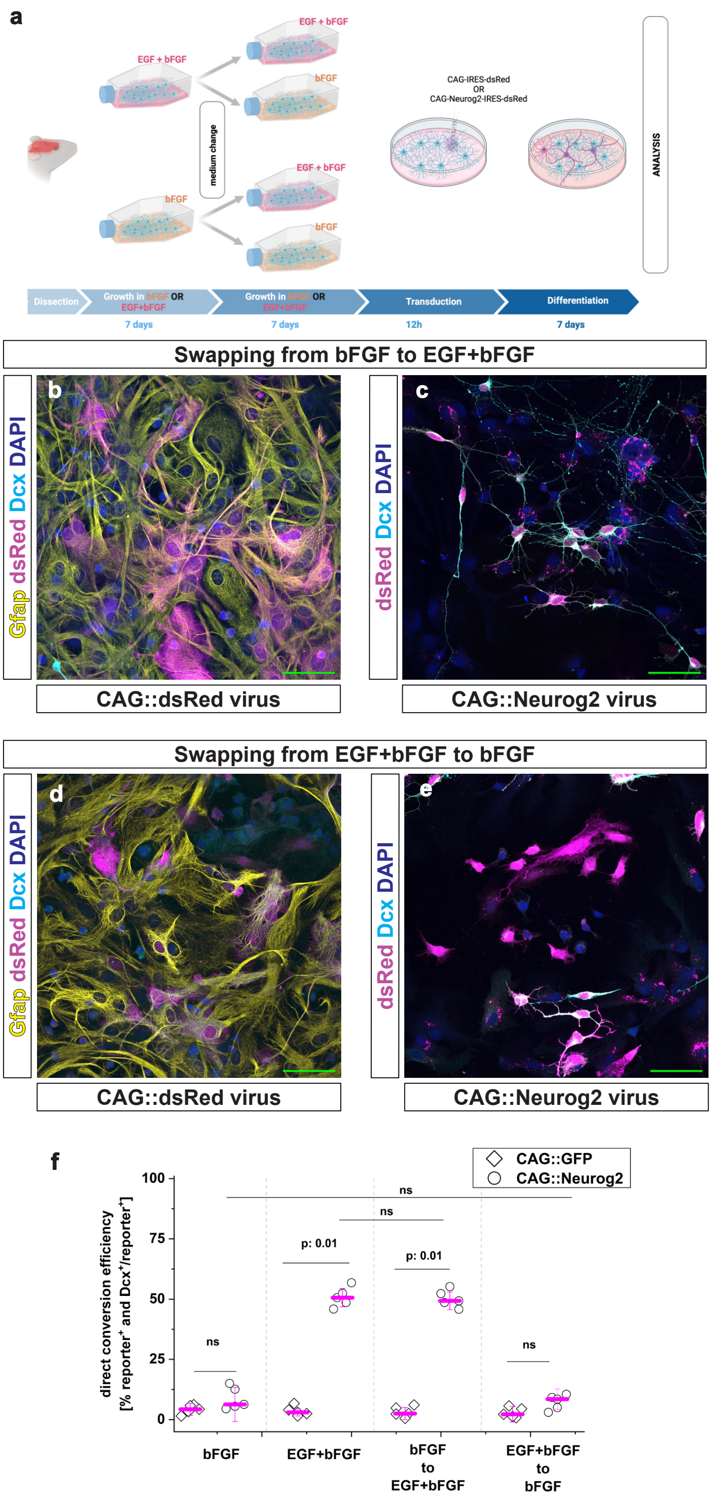

Figure S4: The growth factor induced barrier is reversible.
(**a**) Scheme depicting the experimental paradigm used to address the stability of the growth factor induced lineage barrier. (**b-e**) Micrographs illustrating the identity of control virus (b, d) and Neurog2-encoding virus (c, e) transduced cells cultured first in bFGF and then in EGF+bFGF (b, c), and of cells cultured first in EGF+bFGF and then bFGF (d, e). Identity assessment was performed 7 days after viral transduction. Scale bar in b-e: 50 µm. (**f**) Dot plots showing the proportions of transduced cells acquiring neuronal identity 7 days after viral transduction. Data are shown as median±IQR; single dots represent independent biological replicates. Significance was tested with two-tailed Mann-Whitney test.

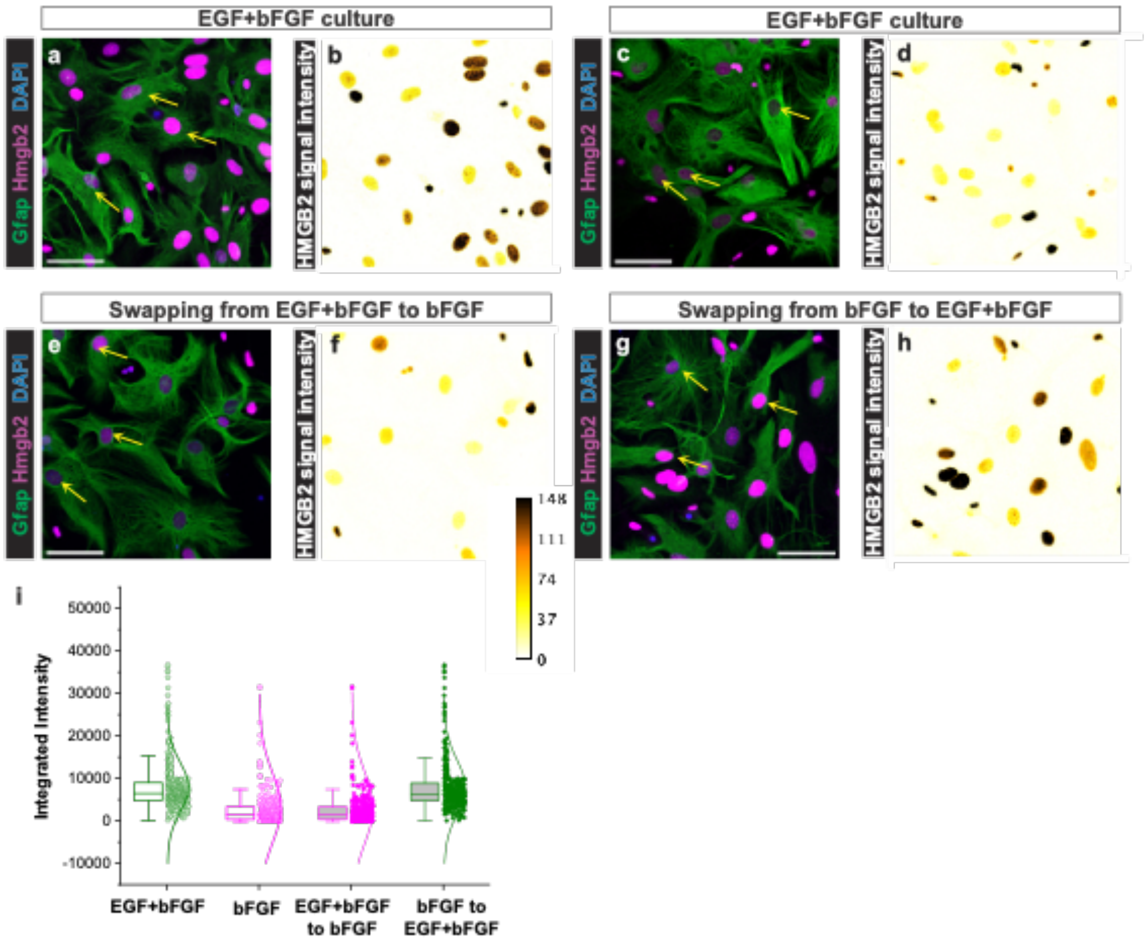

Figure S5: Growth factor induced changes in Hmgb2 expression.
(**a-g**) Micrographs illustrating the expression of Hmgb2 in astrocytes cultured in conditions as indicated in the Suppl. Fig. 4a. b, d, f, h are pseudo colored images depicting expression level of Hmgb2 in single cells. Scale bar in a-g: 50 µm. (**i**) Dot plot showing the expression level of Hmgb2 in single cells. Single dots represent single cells from 4 independent biological replicates.

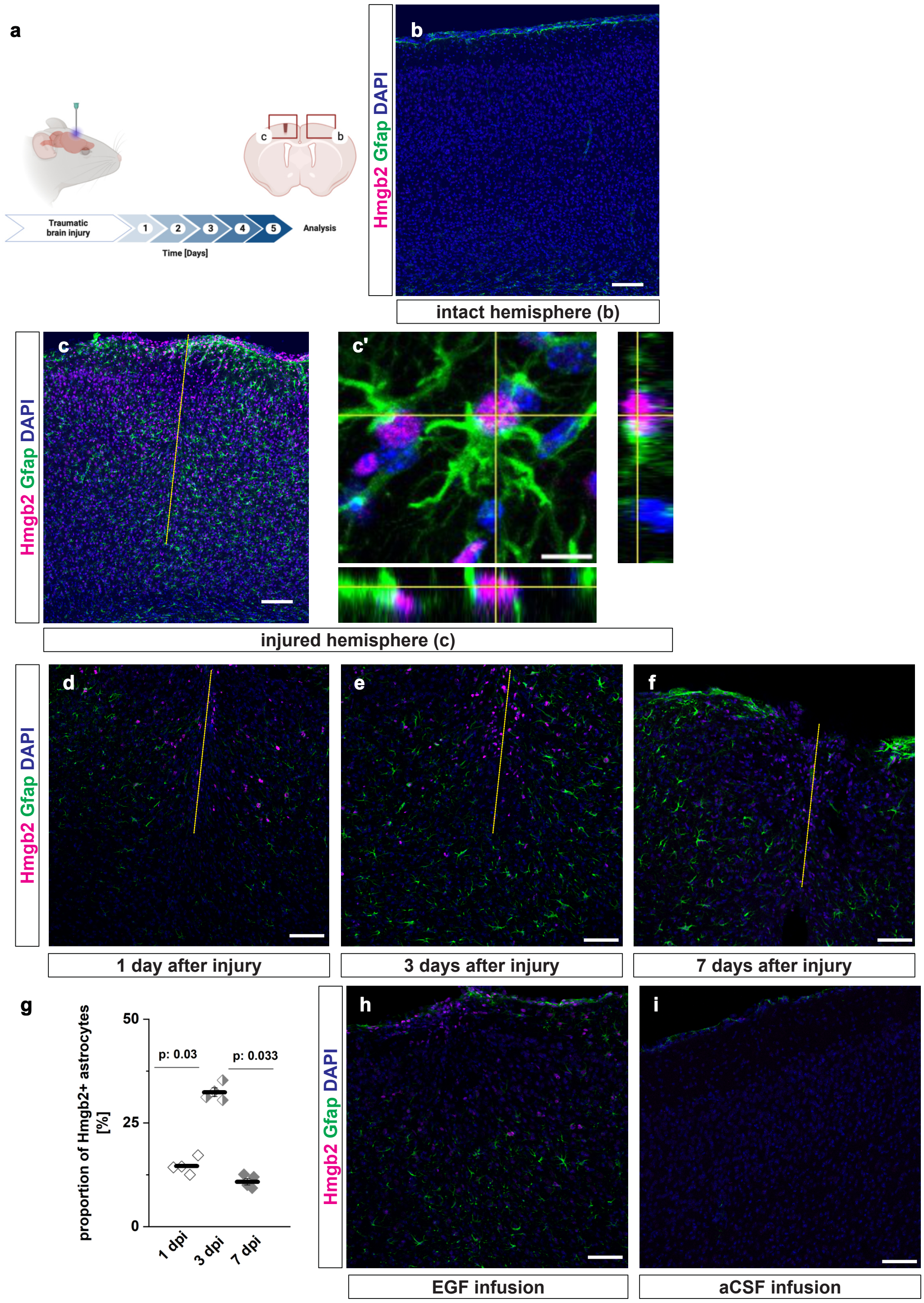

Figure S6: Traumatic brain injury induces Hmgb2 expression in gray matter reactive astrocytes. (**a**) Scheme depicting the experimental paradigm. (**b-f**) Micrographs showing the expression of Hmgb2 in the intact (b) and injured hemisphere (c) at different time points after injury. (c′) Orthogonal projections of the optical Z-stack depicting the expression of Hmgb2 in astrocytes of the injured hemisphere (c). Scale bars in b and c 100 µm and in c′ 10 µm. (**g**) Dot plots showing the proportions of HMGB2 positive astrocytes after injury. Data are shown as median±IQR; single dots represent independent biological replicates. Significance was tested with two-tailed Mann-Whitney test. (**b-f**) Micrographs showing the expression of Hmgb2 following EGF (h) and saline (i) application to the intact brain.

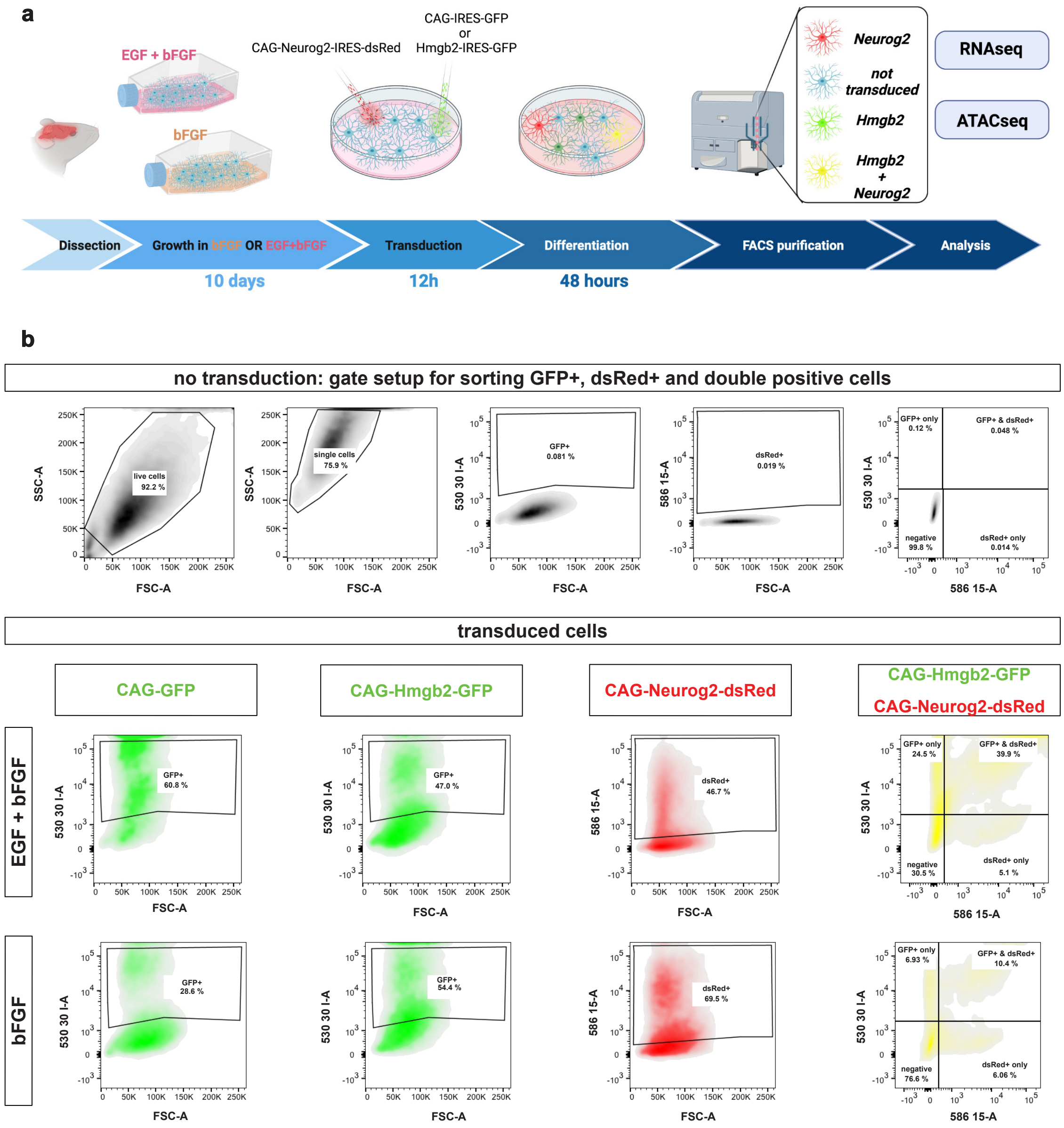

Figure S7: Isolation of transduced cells for RNAseq and ATACseq.
(**a**) Scheme depicting the workflow used to isolate transduced cells 48 h after transduction for omic analysis. (**b**) Plots demonstrating the FACS sorting gates and settings used to sort cells transduced with control, Neurog2 and Hmgb2 expressing viruses.

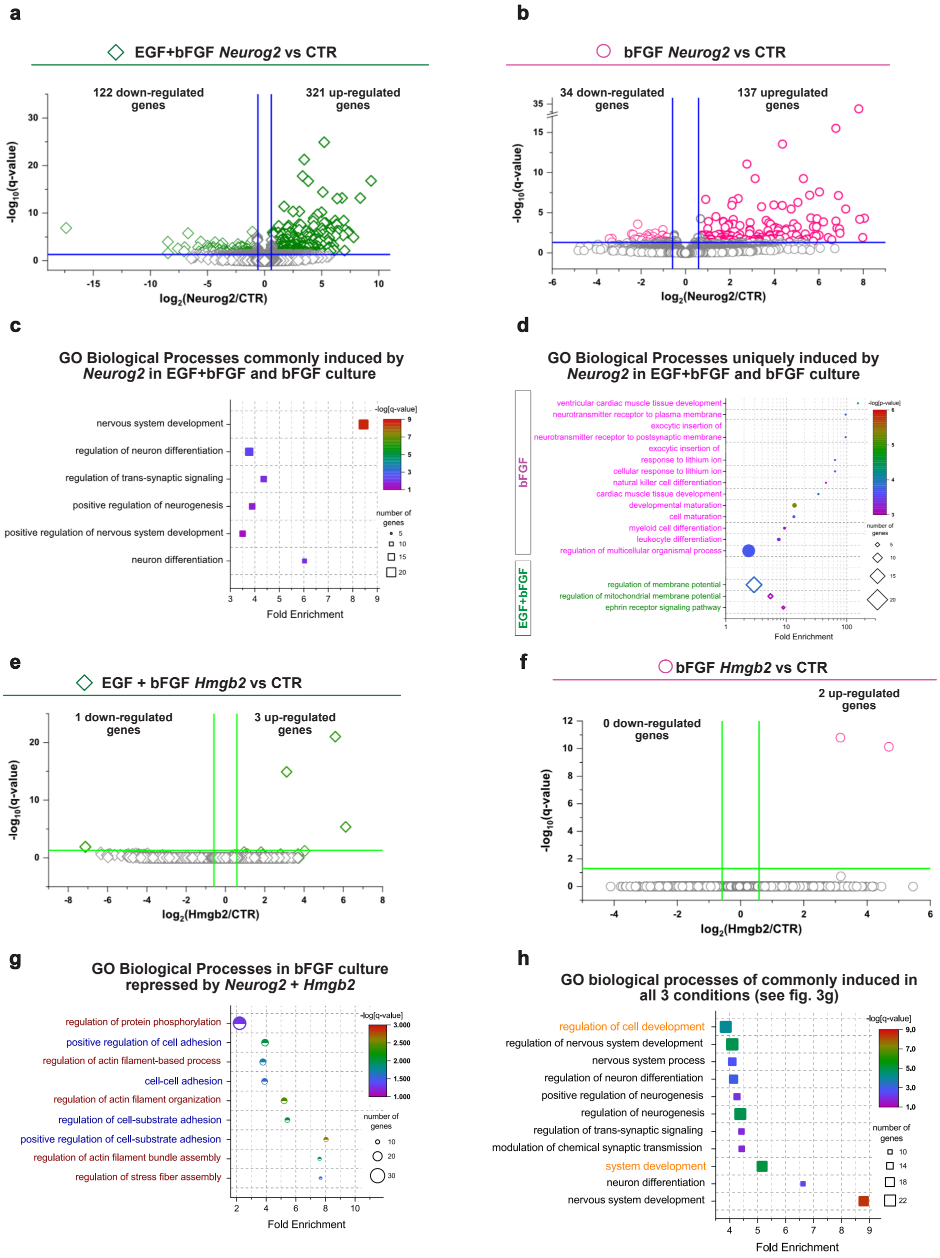

Figure S8. Neurog2+Hmgb2 overexpression in bFGF culture induces a transcriptional subset necessary for successful direct conversion.

(**a-b**) Volcano plots of differentially expressed genes (DEGs) induced by Neurog2 in EGF+bFGF culture (a) and bFGF culture (b) 48 hours after viral transduction. (**c**) Plot depicting enriched GO biological processes of 96 shared genes (Fig. 4c) induced by Neurog2 in both EGF+bFGF and bFGF culture 48 hours after viral transduction. (**d**) Plot depicting enriched GO biological processes of uniquely induced genes by Neurog2 in EGF+bFGF culture (225 gene set; in Fig. 4c, green text) and bFGF culture (41 gene set in Fig. 4c, magenta text) 48 hours after viral transduction. (**e**, **f**) Volcano plot of DEGs induced by Hmgb2 in EGF+bFGF culture (f) and bFGF culture (g) 48 hours after viral transduction. (**g**) Plot depicting enriched GO biological processes of genes downregulated by Neurog2+Hmgb2 overexpression in bFGF culture 48 hours after viral transduction. Red text highlights processes associated with cytoskeletal remodeling, and blue depicts processes involved in adhesion. (**h**) Plot depicting enriched GO biological processes of the gene set commonly induced by Neurog2 in EGF+bFGF, bFGF culture and by Neurog2+Hmgb2 in bFGF culture (88 genes in Fig. 4g). Black text highlights processes associated with neurogenesis.

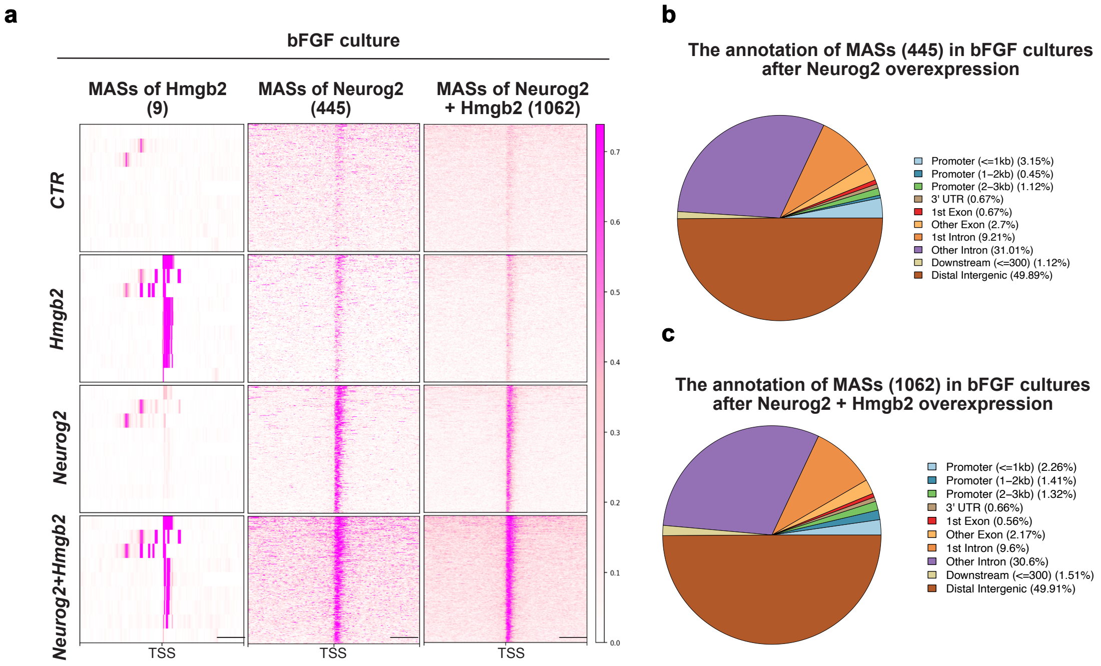

Figure S9. Hmgb2 increases the ability of Neurog2 to open chromatin in bFGF culture. (**a**) Heat map depicting accessibility of MASs induced by Hmgb2 (9 MASs), Neurog2 (445 MASs) and the combination of Neurog2+Hmgb2 (1062 MASs) in bFGF culture 48 h after viral transduction. Scale: 1 kb. (**b-c**) Pie charts of genomic distribution of MASs induced by Neurog2 (b) and the combination of Neurog2+Hmgb2 (c) in bFGF culture 48 h after viral transduction.

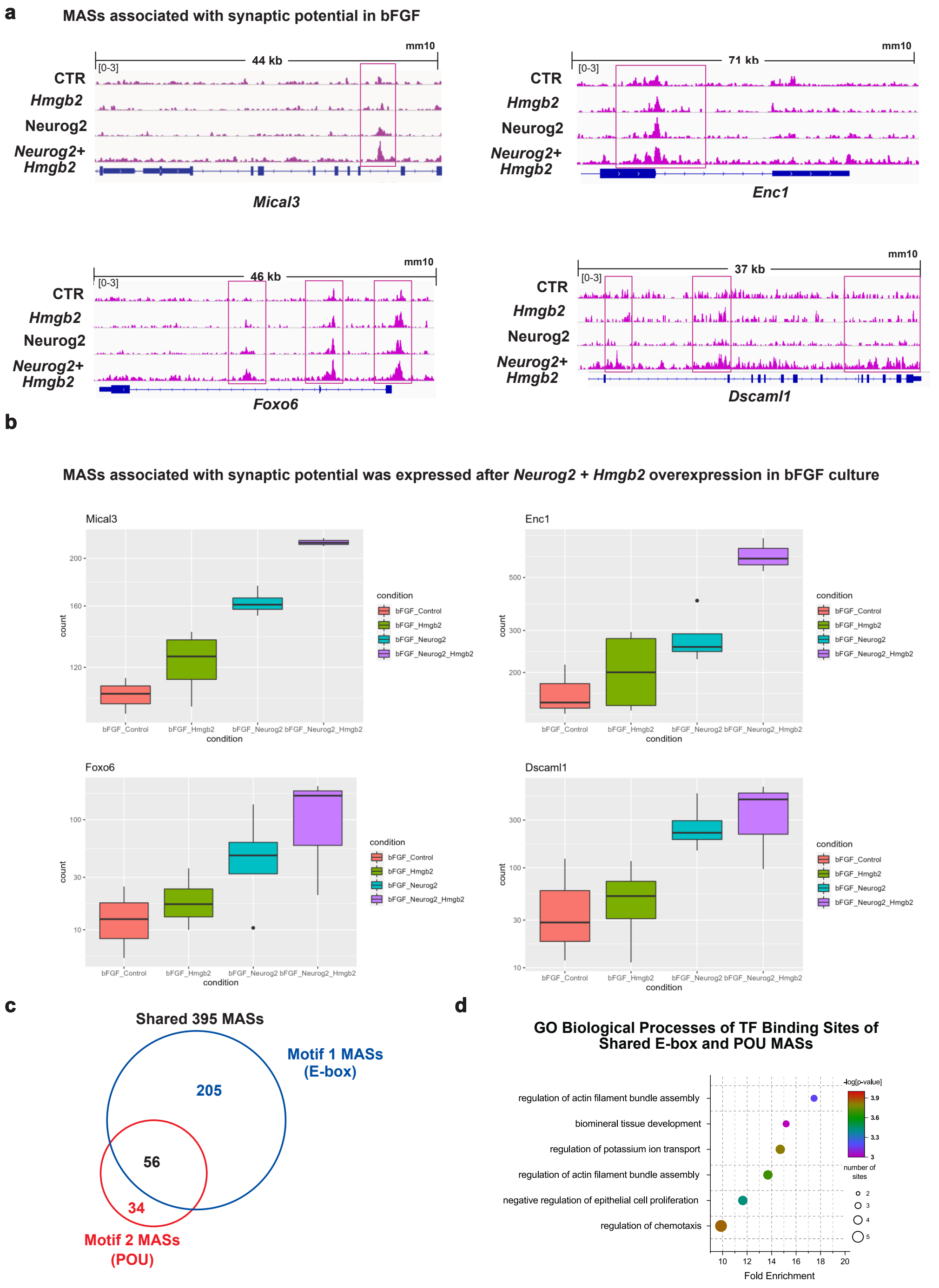

Figure S10. Additional sites opened by Hmgb2 and Neurog2 overexpression are associated with the establishment of synaptic contacts and/or maturation of neurons.
(**a**) IGV tracks showing the ATAC signals of genes associated with synapse formation/function 48 h after viral transduction in bFGF culture. Boxes indicate signals significantly broadened by co-expression of Neurog2 and Hmgb2. (**b**) Box plots depicting expression of synapse-associated genes (from panel a) after control, Neurog2, Hmgb2 and Neurog2+Hmgb2 overexpression in bFGF culture 48 hours after viral transduction. (**c**) Venn diagram illustrating the overlap of MASs with the Tal-associated factor binding motif (motif 1, E-box) and POU domain factor binding motif (motif 2, POU) induced by Neurog2 in EGF+bFGF culture and induced by Neurog2+Hmgb2 in bFGF culture. (**d**) Plot depicting GO biological processes enriched in genes with promoters containing binding motifs for both Tal-associated factors and POU domain factors (56 promoters in c).
