# Supplementary material for: Hmgb2 improves astrocyte to neuron conversion by increasing the chromatin accessibility of genes associated with neuronal maturation in a proneuronal factor-dependent manner
Source: Genome Biol. 2025 Apr 17;26:100. doi: 10.1186/s13059-025-03556-z (PMC12007351; doi:10.1186/s13059-025-03556-z)
Supplement: Supplementary file 9 — Additional File 9: Table S8. List of qPCR primers used for assessment of culture purity [file 13059_2025_3556_MOESM9_ESM.pdf]

Additional File 9: Table S8. List of qPCR primers used for assessment of culture purity.

Gfap
GfapFOR: CAGATCCGAGAAACCAGCCT
GfapREV: ACACCTCACATCACCACGTC
Product size: 146
Tm: 59 ^{o}C

Pax6
Pax6FOR: ACCAGTGTCTACCAGCCAATC
Pax6REV: CAGCATGCACGAGTATGAGGA
Product size: 198
Tm: 58 ^{o}C

Dcx
DcxFOR: TCAGGTAACGACCAAGACGC
DcxREV: AGAGTCATCCAATGACAGCGG
Product size: 150
Tm: 60 ^{o}C

Meis2
Meis2FOR: TTCCAGCATCTCACACACCC
Meis2REV: CCTTGGCTCACTGCTCGATT
Product size: 161
Tm: 59 ^{o}C

Gapdh
GapdhFOR: GTGTTCCTACCCCCAATGTGT
GapdhFOR: ATTGTCATACCAGGAAATGAGCTT
Product size: 248
Tm: 60 ^{o}C
