# Supplementary material for: Hmgb2 improves astrocyte to neuron conversion by increasing the chromatin accessibility of genes associated with neuronal maturation in a proneuronal factor-dependent manner
Source: Genome Biol. 2025 Apr 17;26:100. doi: 10.1186/s13059-025-03556-z (PMC12007351; doi:10.1186/s13059-025-03556-z)
Supplement: Supplementary file 10 — Additional File 10: Table S9. List of ATAC-peaks used for ChIP-qPCR validation including qPCR primers [file 13059_2025_3556_MOESM10_ESM.pdf]

Additional File 10: Table S9: Primers for ChIP-qPCR
List of peaks used for ChIP-qPCR validation with their sequence and primers used in the qPCR. Primer sequence is highlighted in magenta and E-box is indicated by red font.

Git2 - g protein-coupled receptor kinase-interactor 2

chr5:114753431-114753705
CAGAAAAGTCTCGAGATTGGCTGCGAGTTCCCTGAAACCCGCCCTGTTCCAGGCATCGCTC
AGTAAAAACTGCTGAGGAGCTCAGTCCAAGCTCTGTCACTGTCAGCTCCTGGTGGCAGATG
GCAGAGCTGCCCTGAGCCTGGGAGAGGAAGGCAGCCAGGGCTGAGCAGGCCTGCTCTGT
GTGCGGCTCTGGAGGGACCCGGCCTGGCCATCTGCTGAGTTCTGTGTCCTTCCCTAGCTTT
CAGAATACGGATGTCCATCCCAATGACAAAAC
PRIMERS
Peakid33244For: CCAGGCATCGCTCAGTAAAA
Peakid33244Rev: AAGCTAGGGAAGGACACAGAA
Product length: 193

Atp2b2 - atpase, ca++ transporting, plasma membrane 2

chr6:113860622-113861345
AGGGACAGAAGCCTCCAGCCCCAGCATTGTAAGGGACTCCTGGGGAGATTCTCTTTTCAGA
CCCTGGGGCCAGGCCCAGAACAGGAACAGTTGAGGGGCTCTGACTGCCCTGTGGCAGGG
TAGGAGGGGGCGGTGAGCAGATGGCAGCCCCTTACAGTGCTCGAAGAAATCAAGCAGGGG
ATGTGGGAGCTGCCAGCCTGGCCGTATCACCTGCTGTTAGACACAGCCCTGGGTCAGAGG
GCTGGCAGCCACCCAGTACGGATTTGCTGACGAATGGCAGGTCTTGGGGAGGCTCAGCCA
CCAGCTGGCCCTGTTCCATGGCTGCTCTGTTACCTCATTATAAATAAGGGCTGTCACTTGAAA
GGACTCTCCAGTCTAACCCTCCTGGGTCTGTGTCTGTGGAGCATGGAGGGAAGTGACCACT
GCACAGATGAGAAGACTGAGGGTCCAGGAAGAACAGAGATAGGCTTGACAAGGTCACTCAG
GCTTTGTGCTGGGATTGGAGCAAGGCCTGGCTGCCCTGGAGGCTTTGAGGGCGGGACATT
CGAGGAGACGCTGCTGCAGGCGCTCGGGTTCTCTGTGGTGCCAGCTCCCGCTCTGCTACC
ATCTGTCTGCCGGGCTATTTCCGTCTGCCTGAAGTACCCAGGCTCTTGCCCCACCCAGCCG
AGGGCAGAGAACAGAGCCTATGTGGAGACTGTAAGCTCTCCTCAGGTCTCTTTGTGG
PRIMERS:
Peakid35973For: GGCGGGACATTCGAGGAG
Peakid35973Rev: AGGAGAGCTTACAGTCTCCACA
Product length: 174

Tmem108 - transmembrane protein 108

chr9:103709640-103710071
CTTAATACAGATGCAGCTGTaaaggacctttgtagacagttaaagctaagaatgagttgatgtttgagacagtaacattgtca
ggggtgtgtgtgtcccaacaccattatctgagtcctaaaagcaaaattacagatgcatgaagggagggaatccaggaggcggcagca
ggaggagagcgggtgagactaaaagcatgacaggaacttagcctaccactgcaggctgggcagatggaggagggaaagcaatc
caaggaatgtgggcagcttcgaggaggagaatgaccctgctgacagctggcaggaaatgagaatctgggtgtcccaaacacaagac
accagatGCCTCCTGCCCATCTGGTGGATCTGTAAGTATGCCTTCACTTCACCCTAGTGAAATCT
GCCCATTTGTACCAAGAAGTATGAATAC
PRIMERS
Peakid44148For: GCAATCCAAGGAATGTGGGC
Peakid44148Rev: TGGGCAGATTTCACTAGGGTG
Product length: 161

Ntng1 - netrin g1

chr3:109994959-109995745
TCCCGAGATACATTCAGAAAACTATTCAACAATCCCAGCTAATGACTCCAGCTCCAGGAGGCA
CATGTTCAATTAGGACtattttaattttatttttaaaCTGGGAATCTAGATAACCCTGCAATCACTTTTATCT
TCACCAGACCTTGGCGACATAGCAGAGGGACACTGTGGCTTGTGCTAAAGGAACTATGGTG

TGACTGGGGAAACACAAACCTTCATTTGCATGTGAGAGCTCCCCTGGTCGGTACAAATGTCT
CTTCTCTGGCTGTGTGAGCAGTCTGCTTCCTTACTTGGCCTGGAAGCTGGGAGACAATTTTC
TGTGCTTTCTCCTGTAATATTGGAACATTAGGCCCAATTAAAGGCTTCTCCGGAGTTGGCTTT
CCCTGCCTCTTTCTAGCAGAGCCTCCCAAGCTGAAAGAAGAAGCTGCAGAAACAGCGAAGT
GGCAGTGGGGCCAGTAGCAGCAGGCAGTAGTGACAGTATGCCAAGATGAGGATGCTGCGG
ACAGGCTGGCTCTTTGGAAGCTGAAGAAGAGGTGAAGCTGAACAGATGGCAGACCACGCT
GTGTAAGTGTCAGAATGGCTAAGTACACAGACAGCTGCAGAAGGGGTCAGGCTAAGAGAAA
GTACAGAAACAATaaaaaaaaaaaaaaaaaaaaaaaaCAGCAGTGGCTGCCTCTCACCAGCAGTAA
CTGTGACAGAGGCCGTAGCAACAAGAGGCAGCTGAACAGTTAAAGAAGTCAGAGCAGGAAC
AAGGGGAGGTGGGCAGGAGCAGAGAGAAGACA

PRIMERS

Peakid27567For: ACCTTGGCGACATAGCAGAG
Peakid27567Rev: CCAGCTTCCAGGCCAAGTAA
Product lenght: 166

Cplx2 - complexin 2

chr13:54323835-54324597
AATACAGGCTGGCTGTGGAGAGTAGGGCCCCCTCTGGGACACCAGTTGCTCGGTCCTCAAC
TCATTTGGCTAGAATGTAGCGAACCTCAATGTTGTTACCTCCCCTCCCTGTCTATAGTTCTCTG
CTTCTGACTCACTCCCTTATAAACTCGGCTCGCTTCCACCCACAGAAGTTCTTTCAAAGACCA
GGGCTTCCTTCCGAGCTGTCTTTGGTTCCCTTTGACCCATCCTTTCCTCTCAAACCTAAGTCT
TTGTCTGACATCCTTTCTGCTTCCTCTCAGTATTCACCATCAGGGGCTGTAAGGAATAGGATT
TAAAGACCCCCCAGTCTAGATCTGCTGACTGGACTGTCTAAGCCTGTGTCTCAGCACCTATAA
ACAGcctcctggggctgttgtgaaatcaaataagacaaaggatgtggaaatgTGTCCTGCTGTTGCTGAGGATCTT
TGCTGTTAATGAGAGCTCCTTTGACTGCAAATGACAGACAGAGCACAACTACAAAAGGCTTA
AAGGGAAGGCAGTCTGCCATTGCTGGGAACACACGGGTCTGCCCTCCTGTCT**CCATCTG**GT
CTCTACTTGTGCTCCTCACTCTTTGGCCAGCTGTCTAAACCCCCTTTGTACCAGCCAGGAAC
CTGAGTGGAAATGGGCAGCCTGCTTCCCACAGGGCCAGTGGTATTCTGGATTGAACGCCAG
CAGACTAAGCCCTAACCCCATTCCCACAGCCAGGTCCTGGTCACACTCTGTCCCCCAAGGC
TG

PRIMERS

Peakid11157For: AGGCTTAAAGGGAAGGCAGTC
Peakid11157Rev: TTTCCACTCAGGTTCCTGGC
Product lenght: 141

Calb1 - calbindin 1

chr4:15608489-15608913
TTTGAAAGAACACTGCTACCAGACGGTAATATAAAGACCCACTTACCCACTTGGGCAGCCAG
CACAAACAATGAATGTGAATTTCCTGACTAAACAAACATGCTCCAGACTTCCTGCTCTATTGG
AGGGAAGATGCCAAATGTAGGAGGCAGCCATGAATTCTGGCTACTGAAACAGTCCCTTTCAA
TGTGCTGCTCGTCATCTTTCAAAGAACATTTTGTAATCATCGCTGACAGCTGCCTTCCAGAAG
GGAAACAATGAAACATTTCAAAACAGTAGCTTTTGGAAGCAAACTGTAACAGCTTGACACTTG
TGAAGAAGTTCATGGTCCTTGGATATCTACCATCTTGTGAAAAGAGGGACAGCAACTGCTCT
GCTCAGACCCAAATATTTTATAAACTCCCTTACAAAAACTAGGGCTTGT

PRIMERS

Peakid28683For: TCAATGTGCTGCTCGTCATCT
Peakid28683Rev: CAGAGCAGTTGCTGTCCCTC
Product lenght: 193

Nrxn1 - neurexin i

chr17:90704864-90705148
CAGAATGTGTATATGTTCCCTCCCACACGGAGATAGGAATTATGCACACGTCTGATGCAGTCA
GCTGGATGGTTAATTATTCCCTCTGCTTCCCAAGCAGATGGTAGGGCTGGCTCCTCTGAGGC
TGATAAAAGTTCCAGAACAACATCACAGGAGCAGCATGCAAATGAAGTCTCAACATTCTCAAT

GTGGCTCAGTATAATTAGAAGTTTCAAAGTGGATCCCACCACATGCCAGACCGATCAAACAGA
AATATGGCCATTTGGAAAGAGCAGCCTTCTCCC

PRIMERS

Peakid19488For: TGCTTCCCAAGCAGATGGTAG

Peakid19488Rev: TGATCGGTCTGGCATGTGG

Product lenght: 159
